# Supplementary material for: Identification of photoperiod-induced specific miRNAs in the adrenal glands of Sunite sheep (Ovis aries)
Source: Front Vet Sci. 2022 Jul 22;9:888207. doi: 10.3389/fvets.2022.888207 (PMC9354845; doi:10.3389/fvets.2022.888207)
Supplement: Supplementary file 1 [file Data_Sheet_1.ZIP › Supplementary file/miRDeep2 file.zip!/miRDeep2 file/Know_Structure/LP42A3/oar-mir-496.pdf]

miRBase precursor : oar-mir-496
Total read count : 21
oar-miR-496-5p read count : 1
oar-miR-496-3p read count : 18
remaining reads : 2

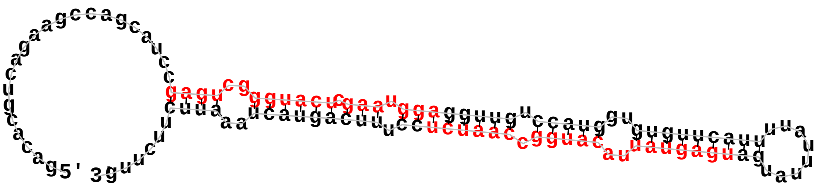

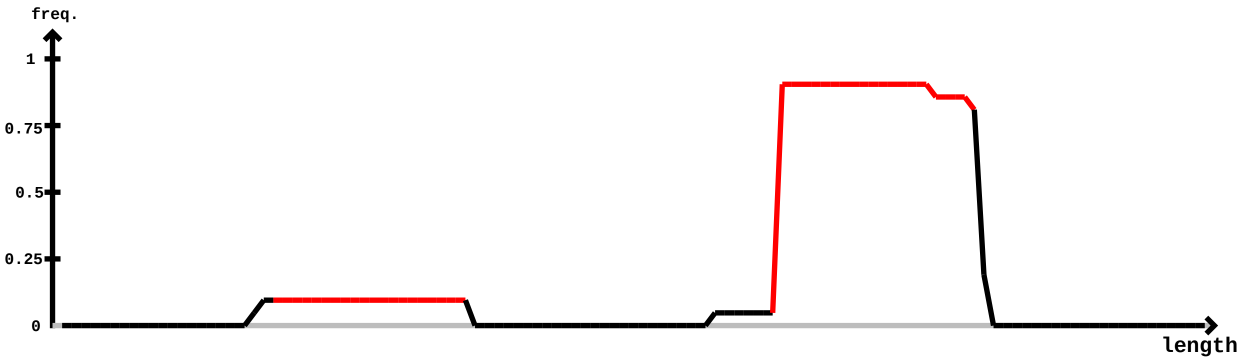


```
                                   oar-miR-496-3p
        oar-miR-496-5p
5'- gacacgucagaagccagcauccgagucggguacucgaauggaggUUGUCCAUGGUGUGUUCAUUUUAUUUAUGAugaguauuacauggccaaucuccuuucaguacuaaauucuucuug   -3'    exp
    ......................((((..((((((.(((.((((((((.(((((..(((((((((........)))))))))..))))).)))))))).)))))))))..))))......   reads     mm    sam
    ...................uccgagucggguacucgaaugga.............................................................................   1         0     sec
    ....................ccgagucggguacucgaaugga.............................................................................   1         0     sec
    ...................................................................uuuaugaugaguauuacauggcc.............................   1         0     sec
    ..........................................................................ugaguauuacauggccaauc.........................   1         0     sec
    ..........................................................................ugaguauuacauggccaaucu........................   13        0     sec
    ..........................................................................ugaguauuacauggccaaucuc.......................   4         0     sec
```
